# Histological Evaluation of Harvested Specimen of a Patient Who Underwent Unicompartmental Knee Arthroplasty Two Years and Six Months After Medial Meniscus Posterior Root Tear Transtibial Pull-Out Repair

**DOI:** 10.7759/cureus.14013

**Published:** 2021-03-20

**Authors:** Tatsuya Kubo, Keiichi Hagiwara, Tsuneari Takahashi, Masashi Kimura, Katsushi Takeshita

**Affiliations:** 1 Orthopaedics, Gunma Sports Medicine Research Center, Zenshukai Hospital, Maebashi, JPN; 2 Orthopaedic Surgery, Jichi Medical University, Shimotsuke, JPN; 3 Orthopaedic Surgery, Zenshukai Hospital, Maebashi, JPN

**Keywords:** medial meniscus posterior root tear, transtibial pull-out repair, histological evaluation

## Abstract

Transtibial pull-out repair is routinely performed to treat medial meniscal posterior root tear (MMPRT). However, data on the postoperative histological evaluation of the repaired medial meniscus posterior attachment after the procedure is scarce. In this report, we present a histological evaluation of the harvested specimen of a patient who underwent unicompartmental knee arthroplasty (UKA) approximately two years and six months after MMPRT transtibial pull-out repair.

The patient was a 75-year-old female. Her X-ray showed Kellgren-Lawrence classification grade II and her MRI revealed MMPRT. Arthroscopic transtibial pull-out repair was performed two months after the onset, and her condition was fine two years after the operation. However, her knee pain gradually worsened, and UKA was performed two years and six months after the initial surgery. The medial meniscus posterior root was continuous from the resected tibia. Tissue specimens were prepared and evaluated.

There were Sharpey's fiber-like tissues in the tibial bone tunnel. The medial meniscus posterior attachment showed a four-layer structure of ligaments, uncalcified fibrocartilage, calcified fibrocartilage, and subchondral bone zone. The structures were observed 2,000 ㎛ medially from the bone tunnel.

The results revealed that the reconstructed graft after a transtibial pull-out repair for the medial meniscus posterior root showed different histological findings compared with the native posterior root and similar to the anterior root of the medial meniscus.

## Introduction

Transtibial pull-out repair is frequently performed to relieve the symptoms of medial meniscal posterior root tear (MMPRT) and to prevent its progression to knee osteoarthritis [1,2]. The procedure has been described to improve clinical scores and prevent the progression to knee arthritis in the short term. However, as per reports, medial meniscal extrusion (MME) did not improve after surgery, and it remains uncertain whether a progression to knee osteoarthritis could be prevented after longer-term follow-up [3-7]. Therefore, surgical methods have been modified to include procedures such as stitches to improve the results, and factors related to poor results are being investigated to clarify the true indications for surgery [8-12]. Currently, data related to the postoperative histological evaluation of the repaired medial meniscus posterior attachment after the transtibial pull-out repair is scarce. We present a histological evaluation of the harvested specimen of a patient who underwent unicompartmental knee arthroplasty (UKA) two years and six months after MMPRT transtibial pull-out repair.

## Case presentation

A 75-year-old female presented with right popliteal pain for three weeks. She had first noticed pain in her right popliteal fossa when she had stepped on her bike; it had failed to improve spontaneously, and she had been referred to our hospital. She had a history of myasthenia gravis and hypertension, both well controlled for over 10 years. She had not injured her right knee during any sports activities. Her body mass index (BMI) was 36.8 kg/m^2^. Initial physical examinations showed effusion of the right knee, tenderness in the posteromedial aspect of the knee, and deep flexion pain. McMurray test conducted when her right knee was externally rotated was positive.

**Figure 1:**
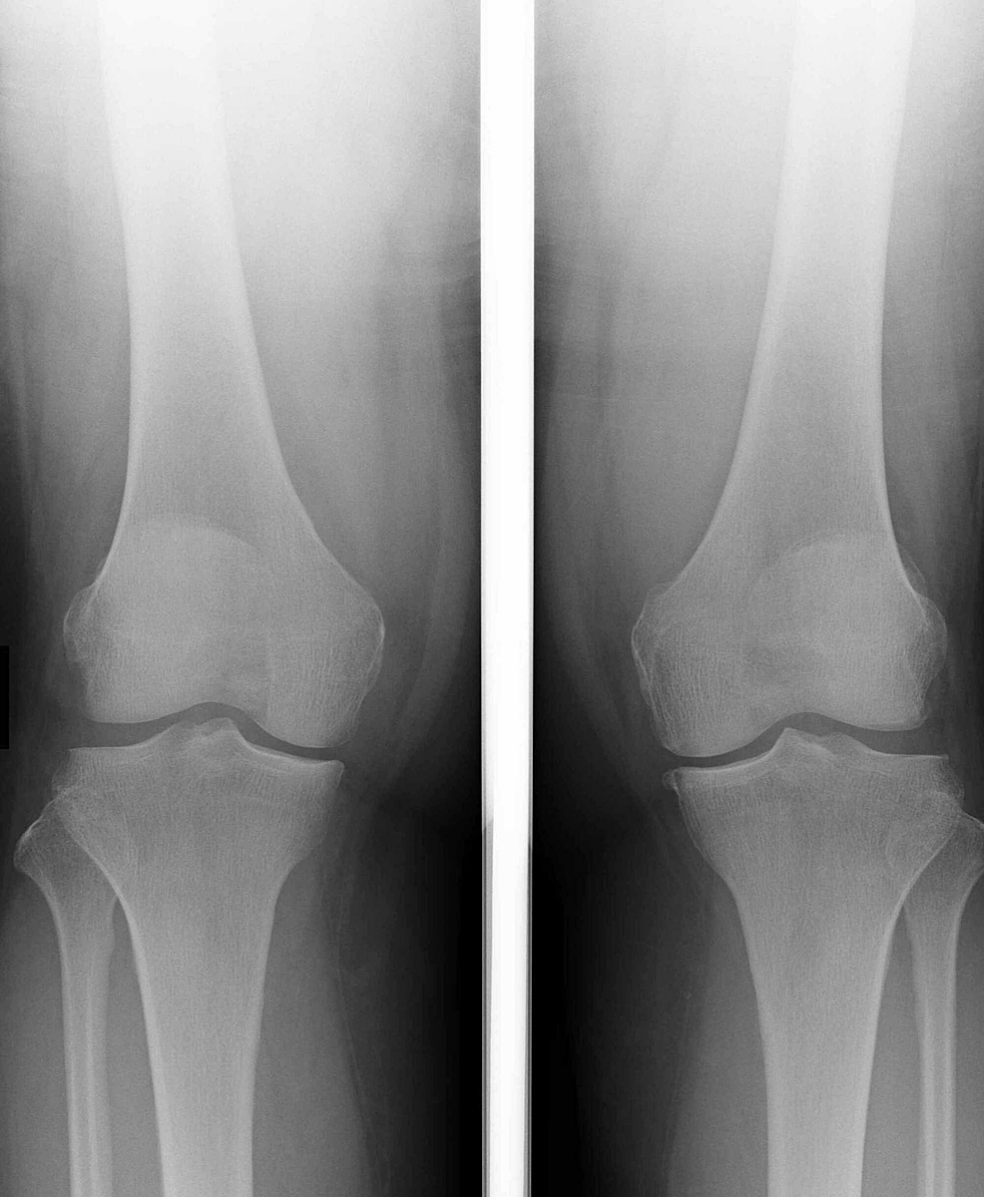
Preoperative X-ray The image showed a femorotibial angle (FTA) of 175 degrees and grade II Kellgren-Lawrence classification

The X-ray showed a femorotibial angle (FTA) of 175 degrees and revealed grade II Kellgren-Lawrence classification (Figure 1).

**Figure 2:**
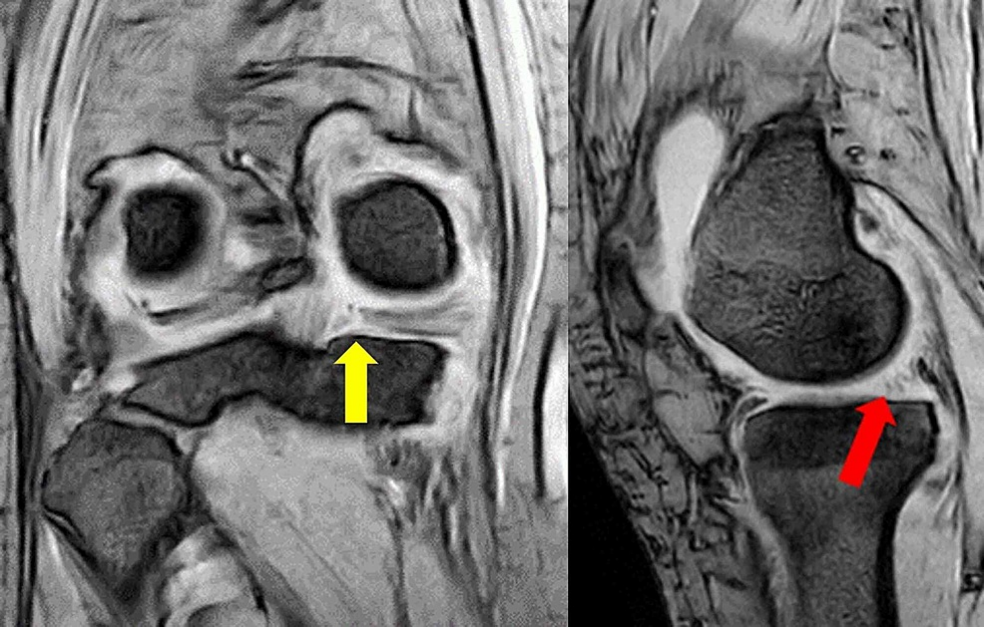
Preoperative MRI T2* (coronal and sagittal views) The images showed truncation sign (yellow arrow) and ghost sign (red arrow) MRI: magnetic resonance imaging

The coronal view of MRI showed a 6.3-mm extrusion of the medial meniscus and a truncation sign, and the sagittal view showed a ghost meniscus sign (Figure 2).

Based on the above findings, a diagnosis of MMPRT was reached. Arthroscopic transtibial pull-out repair was performed two months after the onset of the condition.

**Figure 3:**
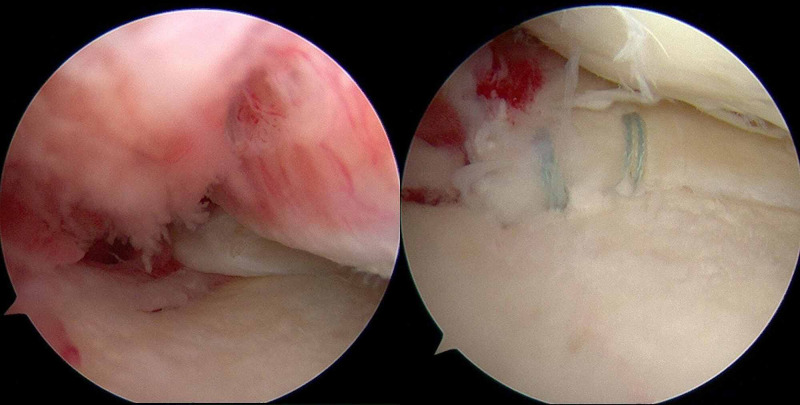
Arthroscopic findings The arthroscopic evaluation found LaPrade classification Type 2A MMPRT. A pull-out repair was performed for MMPRT MMPRT: medial meniscal posterior root tear

A routine arthroscopic evaluation found LaPrade classification type 2A MMPRT and International Cartilage Repair Society classification grade 3 cartilage injury on the medial femoral condyle (Figure 3).

After the preparation of a bony bed by resecting the cartilage around the insertion site of the posterior horn of the medial meniscus, the anterior cruciate ligament (ACL) reconstruction tibial guide with posterior root aiming device was inserted, aimed at the attachment site of the medial meniscus posterior root. A 20-mm socket-shaped bone tunnel with a diameter of 6 mm was created. Two loop stitches using ULTRATAPE® (Smith & Nephew Endoscopy, Andover, MA) and No. 2 FiberWire 5-mm medial from the torn edge in a horizontal direction were performed. By pulling the ends of the suture materials under adequate tension through the tibial tunnel, the meniscus was reduced and stabilized. Suture ends were then tied over an Endobutton® (Smith & Nephew Endoscopy, Andover, MA) and placed underneath the periosteum overlying the anteromedial tibial cortex.

**Figure 4:**
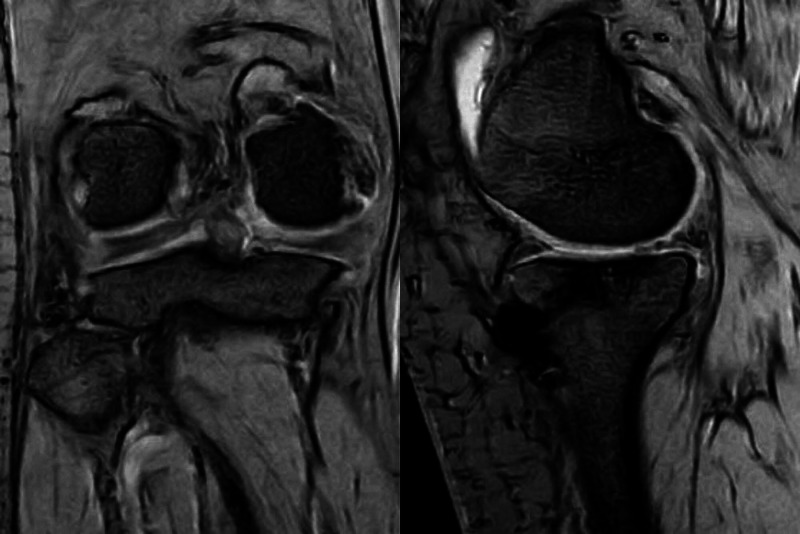
Postoperative MRI (two years later) Medial meniscus posterior root was observed to connect to the insertion site MRI: magnetic resonance imaging

A long leg splint was applied to the knee for immobilization at a slightly flexed position for four weeks postoperatively. The weight-bearing training was permitted four weeks after the surgery. The patient recovered to pre-injury level after rehabilitation and her Lysholm score improved from 55 points to 82 points and the International Knee Documentation Committee subjective score also improved from 27 points to 60 points two years after the surgery. On the other hand, X-rays showed a progressive formation of osteophytes around the medial joint space. MRI showed that the posterior root of the medial meniscus was continuous from the attachment; however, the width of the MME had increased from 6.3 mm to 9.6 mm (Figure 4).

**Figure 5:**
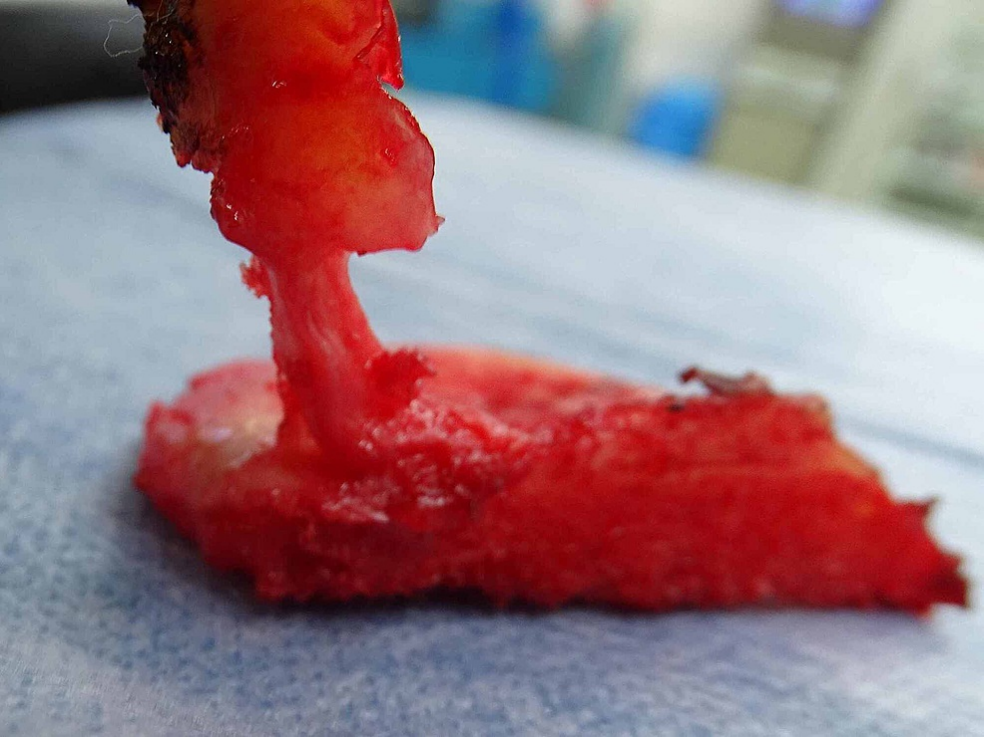
The resected proximal tibia and medial meniscus posterior attachment site at the time of the surgery Medial meniscus posterior root was observed to connect to the insertion site

The patient subsequently had several falls, and the right knee pain gradually got worsened with protective limping. Therefore, UKA was performed two years and six months after the initial surgery (Figure 5). The posterior root of the medial meniscus was continuous from the attachment and was included in resected proximal tibia at the time of the surgery.

## Discussion

The retrieved specimens were fixed by using a 10% neutral buffered formalin solution for 24 hours at 4 °C. Then, 4-μm-thick sections were cut in the longitudinal plane of the posterior root and stained with hematoxylin and eosin for histomorphological observation. The cartilage matrix was evaluated using Toluidine blue staining. Specimens of patients with total knee arthroplasty (TKA) of the same age without MMPRT were prepared and used as controls. The sections were evaluated using light microscopy (BIOREVO BZ-9000; Keyence Corp., Itasca, IL).

**Figure 6:**
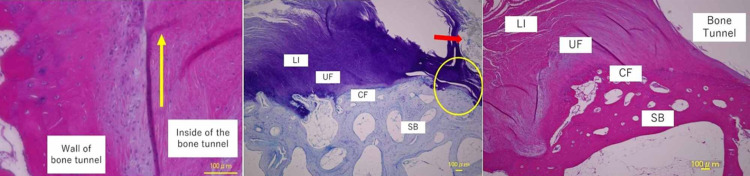
Repaired case (H&E stain ⅹ100; Toluidine blue stain ⅹ100; H&E stain ⅹ100) Sharpey's fiber-like tissue (yellow arrow) was found in the bone tunnel (left figure). The middle figure shows sutures (red arrow) found in the bone tunnel (yellow circle). The meniscal attachment showed the fibrocartilaginous enthesis as the ligament-uncalcified, fibrocartilage-calcified, fibrocartilage-subchondral bone LI: ligament zone; UF: uncalcified fibrocartilage; CF: calcified fibrocartilage; SB: subchondral bone; H&E: hematoxylin and eosin

Sutures on the meniscus were found intact in the bone tunnel, and there were Sharpey's fiber-like tissues in the tibial bone tunnel [13]. The medial meniscus posterior attachment showed a four-layer structure of ligaments, uncalcified fibrocartilage, calcified fibrocartilage, and subchondral bone zone. The structures were observed 2,000 ㎛ medially from the bone tunnel (Figure 6).

**Figure 7:**
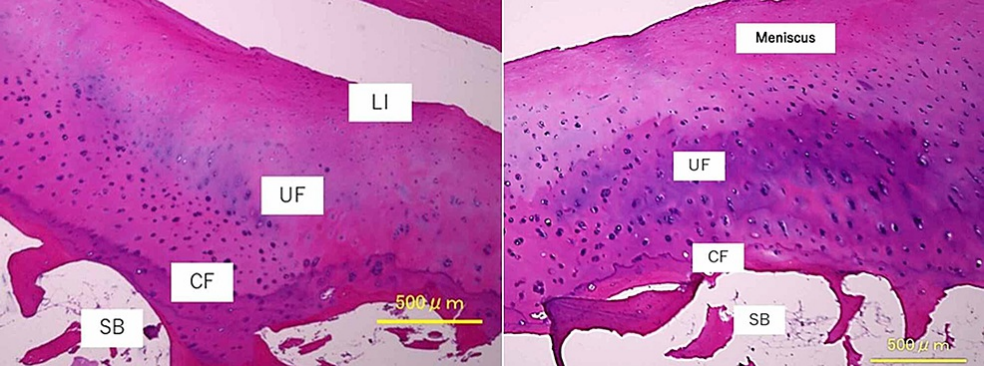
Medial meniscus insertion site of the control case Left figure: anterior insertion site; right figure: posterior insertion site The medial meniscus anterior insertion site has a four-layer structure of ligament, uncalcified fibrocartilage, calcified fibrocartilage, and subchondral bone. On the other hand, the posterior insertion site has a three-layer structure of uncalcified fibrocartilage, calcified fibrocartilage, and subchondral bone LI: ligament zone; UF: uncalcified fibrocartilage; CF: calcified fibrocartilage; SB: subchondral bone

In the TKA patients as controls, the medial meniscus anterior insertion sites were attached via a ligament-like tissue with spindle-shaped fibroblasts and had a four-layer structure of ligament, uncalcified fibrocartilage, calcified fibrocartilage, and subchondral bone. On the other hand, the posterior insertion sites were attached to the tibia without ligament-like tissue, and a three-layer structure of uncalcified fibrocartilage, calcified fibrocartilage, and subchondral bone was observed (Figure 7).

The medial meniscus attaches firmly to the tibia at the insertion site of the anterior and posterior root and has a structure that can withstand contact pressure and hoop stress [14]. The ultimate load of the native human medial meniscus posterior root was reported to be 359 N [15].

The anterior and posterior insertion sites were identified to contain three zones: uncalcified fibrocartilage, calcified fibrocartilage, and subchondral bone. The anterior insertion site contains an additional zone referred to as a ligamentous zone [16]. In our examination, the control case after TKA had a similar histological structure of the insertion site; however, the posterior insertion site of the MMPRT repaired case showed four zones with a ligamentous zone that was characterized by spindle-shaped fibroblasts and collagen fibers on the surface of uncalcified fibrocartilage. This finding was similar to that of the native anterior insertion site and different from native posterior insertion. Regarding our recent literature search, there are currently no reports concerning the postoperative histological evaluation of the repaired medial meniscus posterior attachment. It is unfortunate that we could not compare our results with the histological findings after other surgical procedures for patients with MMPRT.

Since sparse Sharpey's fiber-like tissue was found in the bone tunnel, firm adhesion between medial meniscus pulled into the bone tunnel and cancellous bone was observed to be a well-reconstructed ACL [13]. In addition, the structure of the fibrocartilaginous entheses was observed 2,000 ㎛ medially from the bone tunnel. This indicated that the meniscus after the pull-out repair was fixed to the tibia both at the tibial articular surface near the bone tunnel and inside the bone tunnel. Therefore, postoperative stress distribution around the insertion might be different from the native one and this was a plausible reason for remained MME and re-injury.

Our report has several limitations. First of all, since this case required re-operation, we cannot comment on the postoperative histological findings of patients who do not require any additional intervention and have a good course. Secondly, since we did not perform any biomechanical evaluations, we could not analyze the effects of repair surgery on the attachment structure.

## Conclusions

Based on our findings, the reconstructed graft after a transtibial pull-out repair for the medial meniscus posterior root showed different histological findings compared with the native posterior root and similar to the anterior root of the medial meniscus.
